# Pulp Nodules

**Published:** 1894-03

**Authors:** Charles E. Rhone

**Affiliations:** Bellefonte, Pa.


### Domestic Correspondence.

### PULP NODULES.

BELLEFONTE, PA.

TO THE EDITOR:

I have read with much interest Dr. Kirk's paper on "The Lime Formations in the Pulp-Chamber," as I have had several cases of the kind within the past few months.

All of the patients were strong men, Finlanders, working in one of our lime-quarries. The first one, aged thirty-five, complained of pain in the lower left first molar upon occlusion; tapping with an instrument or heat failed to give any response, but pressure with finger or biting upon a piece of wedging-rubber caused pain. The tooth was perfectly free from caries.

Upon extraction, the pulp was found ossified from the apex to the pulp-chamber, including the fibre connecting the inferior dental branch. This had the character of minute needles, which could be forced upon the inferior dental nerve by pressure upon the ossified pulp in the tooth.

The next case was also a Finlander, aged thirty-five. The tooth, the right superior first molar. This gave no response to tapping or heat, but biting on rubber or pressure produced neuralgic pain. This tooth was also free from caries.

Upon extraction, the pulp-chamber was found filled with nodules. The pulp-canal of the longer of the buccal roots was free from these deposits.

My experience in this direction, while limited, has been confined to patients who have lived on the coarsest kind of food and have done the hardest character of work.

Yours truly,

CHARLES E. RHONE, D.D.S.

[We acknowledge the reception of the specimens described. It is rare to find such complete calcification of the pulp.—ED.]

